# Sudden peroneal nerve palsy in an osteoarthritic knee: a case report

**DOI:** 10.1051/sicotj/2017005

**Published:** 2017-03-10

**Authors:** Vijay Kumar, Mayur Nayak, Tahir Ansari, Rajesh Malhotra

**Affiliations:** 1 Department of Orthopaedics, All India Institute of Medical Sciences New Delhi 110029 India

**Keywords:** Peroneal nerve palsy, Varus arthritic knee, Total knee replacement, Synovial cyst, Decompression

## Abstract

Peroneal nerve injuries have been reported in association with various causes around the knee such as traumatic varus injury, traumatic dislocation, upper tibial osteotomy, knee arthroscopy and total knee arthroplasty. Two instances of varus arthritic knee associated with a peroneal nerve palsy have been reported so far. One presented with gradual onset peroneal nerve palsy that recovered with time and the other with sudden onset peroneal nerve palsy that did not recover. We describe the case of a 63-year-old man who presented with a symptomatic varus arthritic knee and sudden onset peroneal nerve palsy with synovial cysts over the lateral aspect of the knee. We performed a total knee arthroplasty with decompression of the synovial cyst in the same patient. Three months following the surgery the patient was walking pain free with a completely recovered nerve palsy.

## Introduction

Peroneal nerve entrapment is the most common entrapment neuropathy found in the lower limb. It is most commonly seen at the fibular neck where the nerve becomes superficial [1, 2]. The causes for neuropathy can be traumatic such as varus injury to the knee [3], dislocation of the knee, [4] procedures such as high tibial osteotomy, [5] knee arthroscopy [6] and total knee arthroplasty [7, 8].

We describe a case of 63-year-old male presenting to us with a symptomatic progressive varus arthritic knee and sudden onset peroneal nerve palsy. A review of literature showed just one similar case in the past; however, the aetiology and the outcome of the surgery were different.

## Case report

A 63-year-old man presented to Orthopaedics Outpatient Department (OPD), All India Institute of Medical Sciences, New Delhi with a history of pain in bilateral knees for 10 years. He had more pain on the left knee as compared to the right knee. He had developed a sudden onset foot drop in the left lower limb for the last four months.

His pain in the left knee was severe to an extent that he was not able to walk even up to one block. He had to use a walker for ambulation. His activities of daily living such as using a transport, climbing stairs, squatting and sitting cross legged were limited. He also gave a history of recurrent episodes of giving way in the left knee. He developed a localized swelling on the lateral aspect of left knee, which was insidious in onset and progressed gradually over a period of the last one year. He had no history of any trauma to the left knee. He did not have any back pain or any radiating pain in his left lower limb. He had a history of coronary artery disease in the past for which he received treatment in the form of an angioplasty with a cardiac stent.

**Figure 1.**
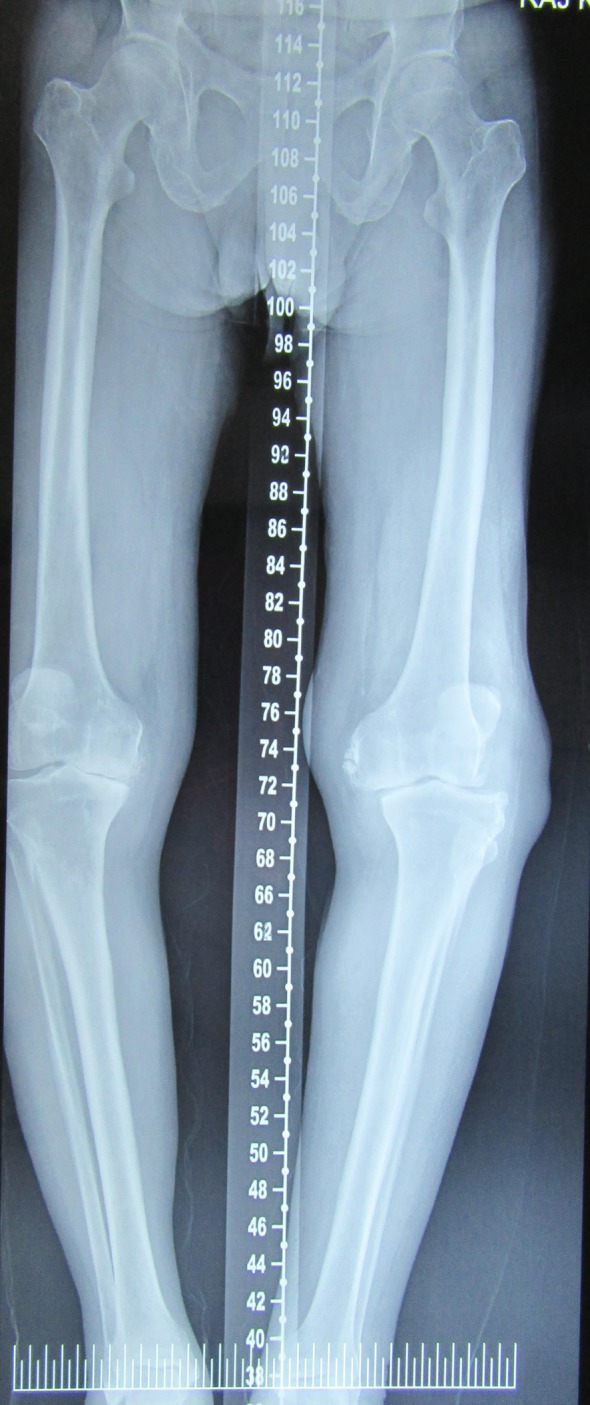
Scannogram of the bilateral lower limb showing varus alignment of both the knees with 10° tibiofemoral angle of the left knee.

**Figure 2.**
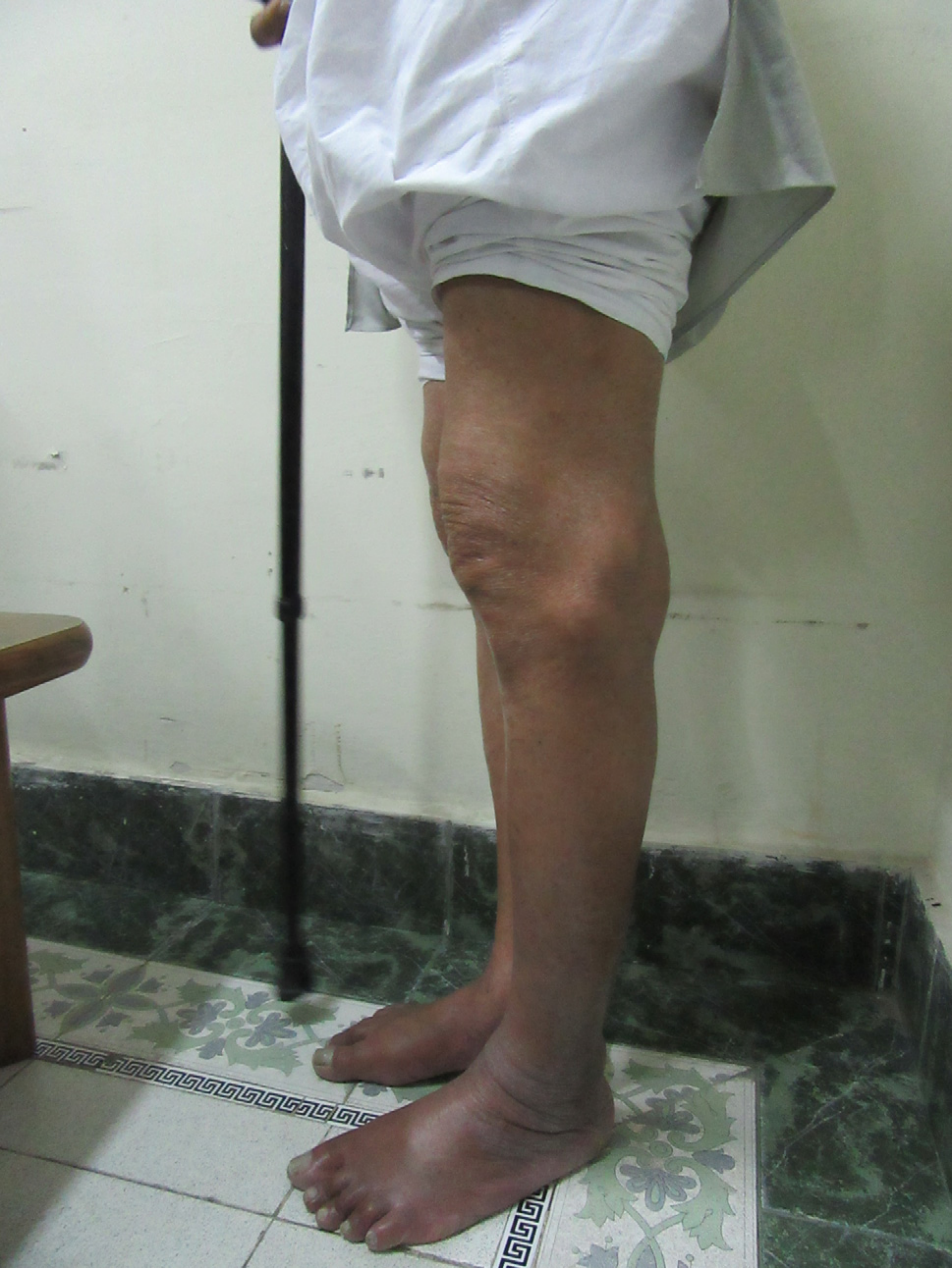
Patient standing with a stick in his hand. From the lateral aspect two swellings can be appreciated. The first swelling was located 1 cm above the lateral joint line and 4 cm lateral from the lateral patellar border and measured 4 cm × 6 cm. The other swelling was located 1 cm below the lateral joint line and 2.5 cm lateral from the lateral patellar tendon measuring 2 cm × 4 cm.

**Figure 3.**
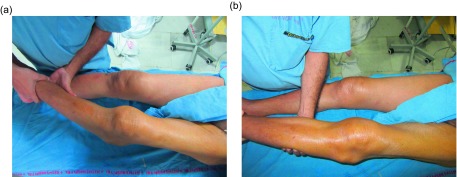
(a) Examination of the ligaments in maximal extension revealed 10 mm of opening of the lateral joint line on varus stress test with a soft end. (b) Examination of the ligaments in maximal extension revealed improvement of the alignment to normal with valgus stress.

On physical examination, the patient was 178 cm tall and weighed 74 kg. He walked with high steppage gait and had a varus thrust. The tibiofemoral angle was 10° varus (Figure 1), on weight bearing. On palpation, the patient had medial joint line tenderness and there was patellofemoral crepitus. There were two swellings located on the lateral aspect of the knee. The first swelling was located 1 cm above the lateral joint line and 4 cm lateral to the lateral patellar border and measured 4 cm × 6 cm. The other swelling was located 1 cm below the lateral joint line and 2.5 cm lateral to the lateral patellar tendon measuring 2 cm × 4 cm (Figure 2). The skin around both the swellings was normal. The patient had 10° of flexion contracture and the range of motion was 10–110° flexion. Examination of the ligaments in maximal extension revealed 10 mm of opening of the lateral joint line on varus stress test with a soft end point and with valgus stress the alignment of the knee improved to normal with a bony end point (Figures 3a and 3b).

Neurological examination revealed a 0/5 motor power in the tibialis anterior and the extensor hallucis longus of the left lower limb. The sensory examination revealed decreased sensation over the dorsum of the left foot and the first dorsal web space. Examination of the hip and the spine revealed no abnormality.

**Figure 4.**
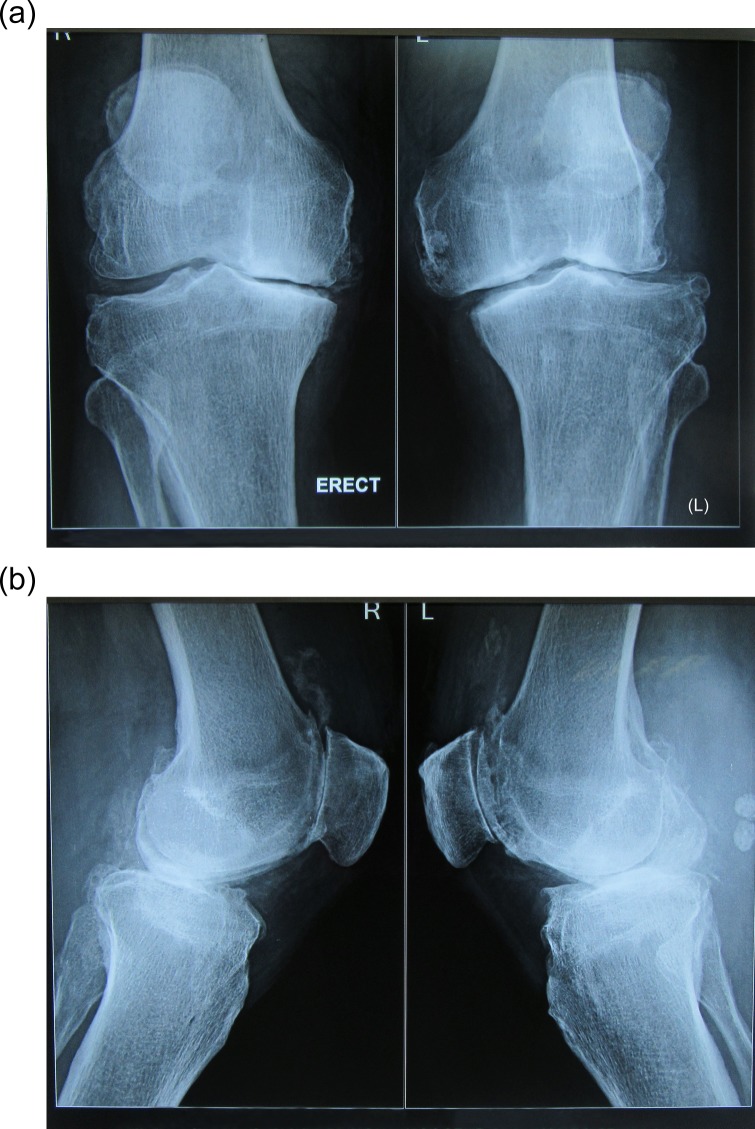
(a) Anteroposterior (AP) X-ray of the bilateral knee showing decrease in the joint space (left > right) with lateral tibial subluxation of the left knee. (b) Lateral radiograph of both the knee showing multiple osteophytes and degenerative changes in the patellofemoral joint.

Radiographs of both the knees’ anteroposterior and lateral views showed tricompartmental osteoarthritis of both the knees with opening of the lateral joint space. There was lateral subluxation of the tibia with respect to the femur (Figures 4a and 4b). Electromyography and nerve conduction study revealed a peroneal nerve neuropathy at the level of the knee.

**Figure 5.**
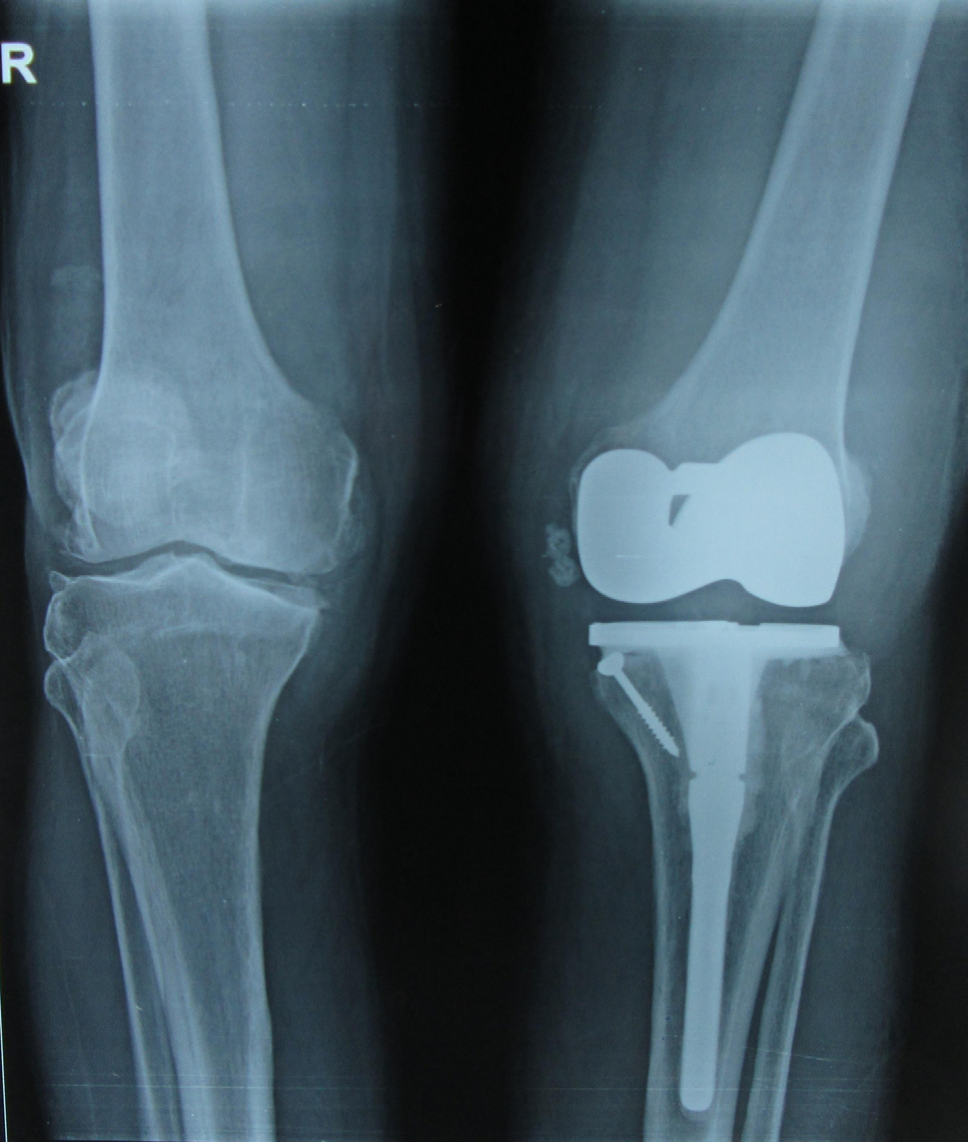
A standing anteroposterior radiograph of the left knee shows a well-fixed, well-aligned, total knee prosthesis with cemented tibial stem and a malleolar screw in the medial aspect.

A total knee arthroplasty (TKA) along with exploration and decompression of peroneal nerve for the left knee was done. A primary TKA (NEXGEN; Zimmer Biomet, Warsaw, Indiana) was performed (Figure 5). Ligament balancing was done by performing medial release. We used posterior stabilized insert with cemented femoral and tibial components with a tibial stem.

**Figure 6.**
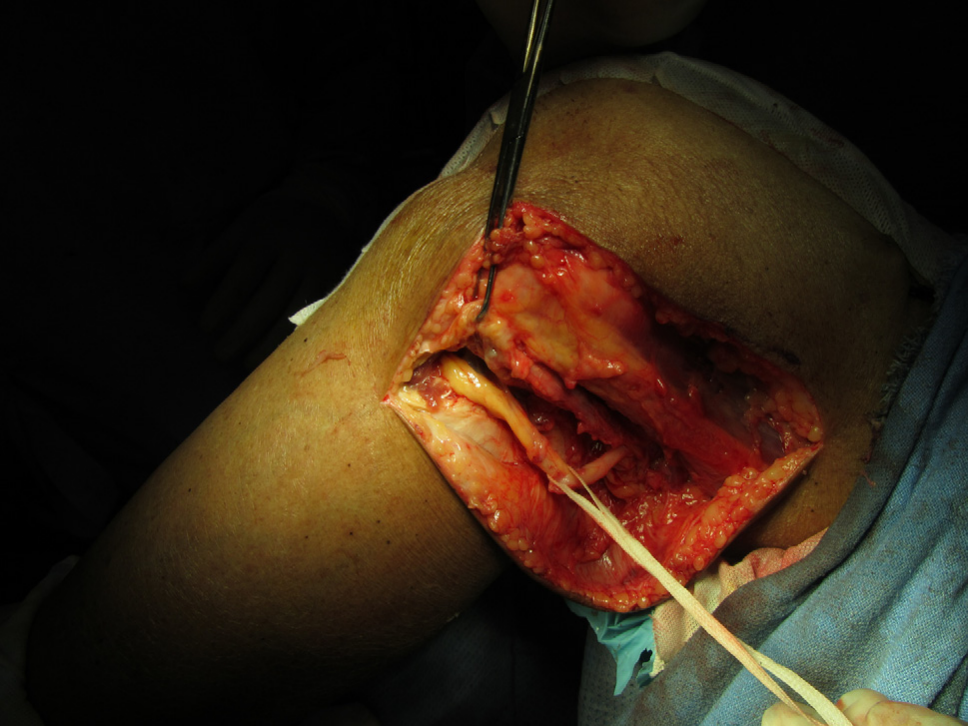
Decompression of the common peroneal nerve at the level of deep peroneal muscles after decompression of the cyst.

**Figure 7.**
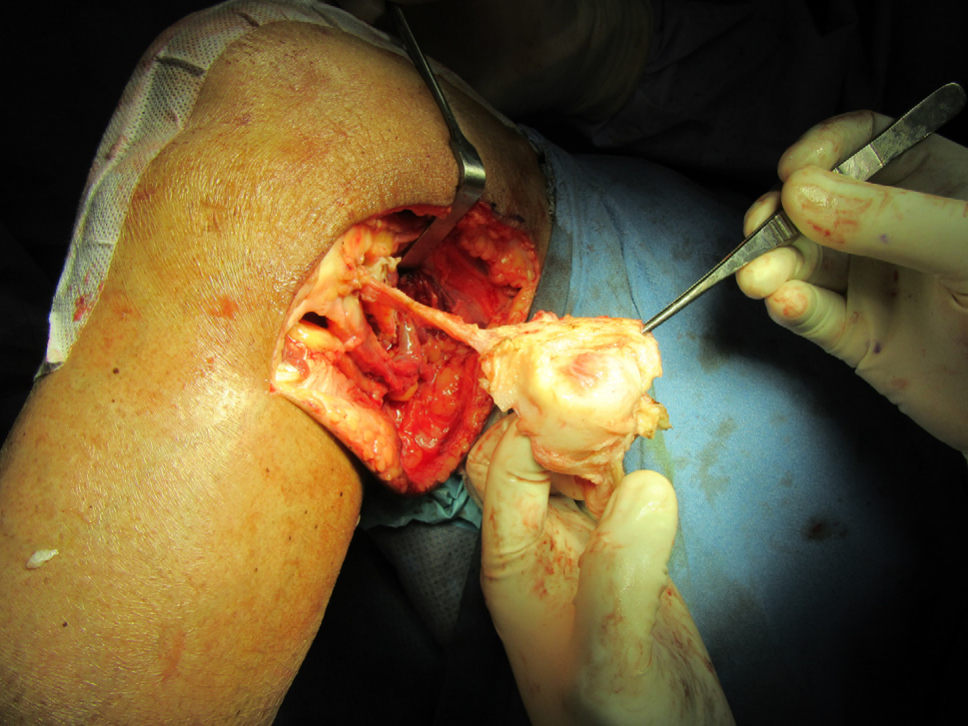
Removal of the cyst in toto. Examination of the inner wall of the cyst reveals smooth and glistening surface.

The peroneal nerve was explored and decompressed by making a 3–5 cm oblique incision parallel to the course of nerve at the neck of the fibula. Skin and the subcutaneous fascia were cut in the same plane. With the help of Metzenbaum scissors and blunt dissection, the retinaculum was identified. The cysts were found under the fascia. Blunt dissection showed cystic wall. An effort was made to remove the cyst without violating the cystic wall, however decompression of the cyst was necessary. Incision of the cyst extruded viscous yellow tinted liquid. The decompressed cyst was then clamped with the Alice forceps and the dissection was started from proximal to the distal directions taking care not to injure the common peroneal nerve around it. The cyst was then removed in toto and was sent for histopathology (Figure 6). The peroneal nerve was found to be embedded between the two cysts and had a flattened surface. The nerve was freed of all the adhesions around it (Figure 7). Furthermore, the nerve was decompressed at the level of deep peroneal muscle fascia after exposing the underlying peroneal muscles.

The patient was made to walk 24 h later with protected weight bearing with the assistance of a walker. The patient was evaluated at two weeks and three months postoperatively. At three months postoperatively, the knee pain has resolved completely and the patient is able to walk and climb stairs unassisted. The power of the left ankle and toe dorsiflexors has improved to 5/5.

## Discussion

The patient presented to us with varus arthritic knee with a four-month history of sudden onset peroneal nerve palsy. Two instances of varus arthritic knee associated with a peroneal nerve palsy have been reported so far. The first report was by Fetzer et al. [9] who reported a patient with varus arthritic knee with gradual onset peroneal nerve palsy. On performing a TKA with peroneal nerve exploration and decompression, the function of the nerve improved. The absence of any compressive lesion at the time of the operation in itself was evident of the fact that the personal nerve dysfunction was due to a tractional injury.

On the other hand, Seyyed Hosseinzadeh et al. [10] reported a patient with varus arthritic knee with sudden onset peroneal nerve palsy. He also hypothesized that the nerve palsy was owing to the tractional injury due to the varus angulation of the knee. However, the nerve was not explored in this case as the injury to the peroneal nerve was believed to be tractional in nature.

In our case, the scenario was similar (varus arthritic knee with sudden onset peroneal nerve palsy); however, there was one difference that the patient had swellings over the lateral aspect of the knee. The disability and the pain were severe enough to warrant a total knee replacement (TKR) of the left knee. The added benefit of this procedure apart from pain relief was that it corrected the varus alignment of the knee, thereby removing any traction to the common peroneal nerve. After performing a TKA in the left knee the cyst was explored and removed, and it was found that the nerve was compressed by the cyst. Synovial cyst can occur in the osteoarthritis of the knee secondary to increased synovial production causing distension of bursa and herniation of the capsule [11]. It is commonly seen in the popliteal and dorsal wrist location. Association of peroneal nerve palsy with synovial cysts has been described in the past by various authors [12]. We presume that this condition was precipitated both by the tractional injury to the nerve as well as compression of the nerve by the cyst. The reason to favour our hypothesis is that after performing TKA and decompression of the cyst with neurolysis, there was full recovery of the peroneal nerve function.

## Conflict of interest

The authors declare no conflict of interest in relation with this paper.
